# Validation of the Updated Digital Health Literacy Instrument and Development of a Short Form: Online Survey Study of the General Population

**DOI:** 10.2196/86879

**Published:** 2026-04-01

**Authors:** Rosalie van der Vaart, Inge Tuitert, Job van 't Veer, Constance Drossaert

**Affiliations:** 1 Centre of Expertise Health Innovation, Technology for Health Care Research Group, The Hague University of Applied Sciences, Johanna Westerdijkplein 75, Den Haag, 2521 EN, The Netherlands, 31 0614188052; 2 Department of Health and Social Studies, NHL Stenden University of Applied Sciences, Leeuwarden, The Netherlands; 3 Department of Psychology, Health and Technology, Faculty of Behavioural, Management and Social Sciences, University of Twente, Enschede, The Netherlands

**Keywords:** digital health literacy skills, eHealth, measurement, validation, instrument, health information

## Abstract

**Background:**

The digital health literacy instrument (DHLI) was developed in 2017 to measure individuals’ ability to access, understand, evaluate, and apply online health information. Since that time, digital health has shifted from desktop-based internet use to mobile devices, and there has been a rapidly expanding range of health apps. Additionally, heightened privacy and data security requirements have increased the complexity of user competencies needed to engage with digital health tools. These developments underscore the need to update the original DHLI.

**Objective:**

This study aimed to create an updated version of the DHLI (DHLI 2.0) that reflects current digital health practices and to examine its reliability and validity by exploring associations with user characteristics. Additionally, we aimed to develop a short-form version to facilitate broader use in research and practice.

**Methods:**

The instrument was iteratively updated and pilot-tested to retain the original theoretical framework while reflecting current digital health practices, devices, and emerging challenges such as mobile use and data security. Several items were reworded and a new 2-item subscale on digital safety was added. The full DHLI 2.0 comprises 24 items across 8 skill domains. A 16-item short form was developed by iteratively removing 1 or 2 items per subscale based on the “α if item deleted” criterion, while retaining the same subscale structure as the full form. Data to validate the new version of the instrument were collected in June 2024 through an online survey among members of a representative citizen panel in Friesland, a province in the Netherlands (N=2728). Sociodemographics, internet and health-related internet use, general health literacy (measured with the Single Item Literacy Screener), self-reported health, and health care use were assessed. Internal consistency was evaluated using Cronbach α, and construct validity was assessed via Spearman ρ correlations with related constructs.

**Results:**

Internal consistency was high for both the full (α=0.94) and short-form (α=0.90) scales. Most subscales showed satisfactory to excellent reliability (α=0.71–0.93), while “Securing privacy” and “Using security measures” demonstrated moderate reliability (α=0.65-0.66). The DHLI 2.0 total scores were approximately normally distributed (skewness –0.5; kurtosis 0.4). As expected, digital health literacy was negatively correlated with age (ρ=−0.39, *P*<.001) and positively correlated with education (ρ=0.22, *P*<.001), income (ρ=0.27, *P*<.001), time spent online (ρ=0.32, *P*<.001), and general health literacy (ρ=−0.42, *P*<.001).

**Conclusions:**

The DHLI 2.0 provides an updated, reliable, and valid measure of digital health literacy covering 8 key domains, including data security. The 16-item short form offers a concise alternative suitable for research and possibly practical applications in health and eHealth contexts.

## Introduction

Over the past decades, the landscape of health care has transformed considerably due to the rise of the internet and digital health technology. Citizens and patients now have access to a broad spectrum of digital health resources, allowing them to search online for health-related information, communicate with care professionals through patient portals, connect with peers via social media platforms, and apply digital tools and applications to monitor or manage their health.

Although these digital health innovations hold great potential to improve accessibility and quality of care and to improve patient involvement in care, not everyone benefits equally from them. For example, data from the Dutch National Monitor Digital Care show stark differences in use of digital solutions among care users, where almost all care users with a theoretical educational background report using a computer independently or with help, compared to about half of those with a practice-oriented educational background [1,2]. These disparities also extend to the use of digital health tools, where these numbers are 68% and 22%, respectively, for using websites to seek health information. In addition, age-related differences are also evident: while 75% of those aged 15 to 39 years reported having used health websites, this is only 36% in the age group of ≥65 years [1,2]. Similar trends have frequently been observed in international research contexts [3-5]. Effectively engaging with digital health resources requires a set of complex skills that are not evenly distributed across the population. People may struggle to access, navigate, understand, evaluate, or apply online health information, or to use digital health tools efficiently.

These findings highlight the importance of digital health literacy, defined as the ability to seek, find, understand, appraise, and apply online health information and services [6]. As health information and care services are increasingly delivered through digital channels, gaining insight into individuals’ digital health literacy is essential. It allows researchers to assess the level of digital health literacy at a population level and provides insight into differences in digital health literacy skills among subgroups. In addition, it supports researchers and developers of health applications to assess whether innovations fit their target group. For health care professionals, knowledge about their patients’ digital health literacy could even be useful to assess whether support is needed, for whom, and in which form. To assess digital health literacy, the digital health literacy instrument (DHLI) was developed in 2017 [7]. The DHLI is grounded in theory and empirical observational research and encompasses 7 subscales. Each subscale captures a distinct component of digital health literacy, and together they represent the practical skills needed to access, understand, evaluate, and apply digital health information: operational skills (the skills to operate a device, such as typing, clicking, and swiping), navigation skills, information searching, evaluating reliability, determining relevance, creating self-generated content (being able to express oneself clearly in written language), and protecting privacy while sharing information with others.

Since its introduction, the DHLI has been widely adopted and validated across national and international contexts, including studies among diverse patient populations [8,9], adolescents [10,11], older adults [12], university students [13-15], and health care professionals [16], demonstrating its value as a comprehensive instrument for assessing digital health competencies. Yet, the digital health landscape has evolved considerably since the publication of the DHLI. First, there has been a shift from desktop computers and laptops to mobile devices, such as smartphones and tablets, which require different operational skills (eg, tapping and swiping rather than clicking or typing). Second, the field has seen a rapid increase in digital health tools (eg, apps for self-management, self-monitoring, and communication), making some of the original DHLI items, which primarily focus on internet use, less suitable. Third, growing concerns around privacy and data safety have led to more complex authentication procedures, such as 2-factor identification and the need to manage secure passwords, which demand additional user competencies [17].

In response to these changes, we felt the need for a revision of the DHLI. Therefore, the first aim of this study was to validate an updated version of the DHLI, the DHLI 2.0, including its internal consistency and construct validity, by examining the associations with a range of user characteristics. Additionally, we aimed to examine the feasibility and validity of a short-form version of the instrument and to facilitate broader applications in research and practice.

## Methods

### Instrument Revision

The revised version of the DHLI, the DHLI 2.0, includes 8 skill domains, each measured with 2 to 4 items, resulting in 24 items in total. The goal of the revision was to keep the original structure and theoretical framework of the DHLI intact, while updating the instrument to current digital health practices and currently used devices. The revision was an iterative process, with some experts, in which we reworded the items to make them more in line with the vocabulary of the current digital landscape. Subsequently, a pilot study was conducted with a preliminary version. The prefinal version of the new instrument was tested in a qualitative pilot study, with 8 survey experts of Planbureau Fryslân, which informed the final adjustments. As main changes, we added the use of tablets and smartphones in the introduction of the items and included words as tapping and swiping (in addition to clicking). A new subscale, including 2 items, was added to address data security challenges based on problems encountered in recent projects. The items include skills to use 2-step authentication and skills in using strong passwords.

Similar to the original version, all items are scored on a 4-point Likert scale, with response options ranging from “very easy” to “very difficult” or from “never” to “often.” For the current validation study, we added an option “not applicable/do not know,” since this was common practice in the surveys of Planbureau Fryslân. For analysis purposes, scores are reverse-coded so that higher values reflect higher levels of digital health literacy. A total digital health literacy score was computed by averaging responses across all items, provided that at least 16 items have been completed in the full scale and 8 items in the short form (SF). Subscale scores were calculated using the mean of the items within each skill domain, provided that all items of the subscale were completed. MultimediaAppendices 1^3^ provide (1) the items and scoring instructions for the revised DHLI, full form (FF DHLI 2.0); (2) the SF DHLI 2.0; and (3) an overview of the changes made in DHLI 2.0 compared to the original DHLI.

### Study Design and Participants

For this study, the citizen panel from Planbureau Fryslân was used. Planbureau Fryslân is the independent regional institute for research and monitoring, aiming to inform and enhance local government policy in the province of Friesland, the Netherlands. Their panel consists of a sample of approximately 8000 residents in Friesland (a Northern province of the Netherlands). To ensure representativeness, Planbureau Fryslân regularly draws a random sample of all residents aged 18 years and older in the province, in close coordination with the municipalities. Potential panel members are personally invited by letter to participate in the sample. Self-enrollment in the panel is not possible. Panel members are periodically asked to participate in surveys on topics relevant to regional policy, including health. The data collection for Panel Fryslân follows a standardized procedure as described in their research accountability document [18]. For this study, in June 2024, all active panel members were invited to complete a survey on digital health care use and digital health literacy. The questionnaire included the DHLI 2.0 and 5 other digital health-related questions (see Measures). Panel members could respond to the survey during a 3-week period and received 2 reminders within these 3 weeks. Three months earlier, in January 2024, the panel had filled out a survey on general health, including questions on self-reported general health, health care use, and chronic health conditions (see *Measures*). These variables were merged with the data from June 2024 for the current analyses. In total, 2728 respondents filled out both the survey from January and June 2024. The data of these respondents are used in the analyses.

### Procedure

Data collection by Planbureau Fryslân was carried out via an online Dutch-language survey (Quest Software, Inc), which was distributed via email to panel members. Panel members were given 1 month to respond to the survey and received 2 reminder emails during that period to enhance the response rate.

### Measures

In addition to the DHLI 2.0, the survey items addressed the following characteristics of the participants: (1) sociodemographics (gender, age, educational attainment, and income); (2) internet use (average number of hours spent online per day); and (3) health-related internet use (the use of 11 different online health activities in the past 12 months) (see Table 1 for a complete overview of all activities); with these 11 items, a sum score was calculated (ranging from 0 to 11), where using 1 specific type of online health activities added 1 point to the sum score; (4) general health literacy (assessed using the Single Item Literacy Screener [SILS]) [19]; (5) self-reported general health on a scale from worst possible health (1) to the best possible health (10); (6) health care use (assessed by number of visits to a general practitioner in the past 12 months, visits to a medical specialist in the past 12 months, and visits to a mental health professional in the past 12 months); and (7) presence of chronic conditions.

**Table 1.** Internet use and health-related internet use (N=2728).

Variable	Value
Hours spent online per day, n (%)	
<1	371 (13.6)
1‐3	1346 (49.3)
3‐5	551 (20.2)
5‐8	237 (8.7)
>8	148 (5.4)
Not applicable/don’t know	35 (1.3)
Missing	40 (1.5)
Self-rated internet skills, n (%)	
Very good	624 (22.9)
Good	1186 (43.5)
Average	536 (19.6)
Less than average	272 (10)
Poor	50 (1.8)
Not applicable/don’t know	20 (0.7)
Missing	40 (1.5)
SILS^a^, n (%)	
Never	1692 (62)
Rarely	739 (27.1)
Sometimes	218 (8)
Often	29 (1.1)
Always	3 (0.1)
Not applicable/don’t know	7 (0.3)
Missing	40 (1.5)
Number of respondents who have used the internet for health-related reasons	
Online information, n (%)	2353 (86.2)
Accessing a patient portal^b^, n (%)	1729 (63.4)
Online prescriptions, n (%)	1220 (44.7)
Accessing a personal health portal^c^, n (%)	1175 (43.1)
Wearable, n (%)	1027 (37.6)
Online appointment scheduling, n (%)	855 (31.3)
Information on a health app, n (%)	824 (30.2)
Video consultation, n (%)	428 (15.7)
Information on social media, n (%)	423 (15.5)
Healthy lifestyle app, n (%)	367 (13.5)
Communicating via a patient forum, n (%)	170 (6.2)
Health-related internet use, mean (SD)	3.93 (2.24)

a SILS: Single Item Literacy Screener.

b Data from a care provider shared via a secured portal.

c Health data from care providers combined on a personal secured portal.

### Ethical Considerations

All participants provided informed consent upon joining the panel and agreed to the use and sharing of their anonymized data for research purposes. The panel adheres to the code of conduct established by the Dutch Association for Statistics and Research (Vereniging voor Statistiek en Onderzoek, VSO) [20]. This study was reviewed by the Ethical Board of NHL Stenden University of Applied Sciences, who determined that the study is indeed in line with this conduct (reference number Tuitert 202405). Prior to being shared with the authors of this paper, the data were fully anonymized. The authors had no direct contact with the participants, and no financial compensation was provided for participation in the panel.

### Data Analyses

Data were analyzed using IBM SPSS version 29.0 for Windows (IBM Corp). Descriptive statistics were computed for all relevant variables, including frequencies, percentages, means, and SDs. Missing values and the option “don’t know/don’t want to tell” are in most cases reported in the descriptive statistics but were treated as missing values in further analyses.

Cronbach α served as a measure of internal consistency, reflecting the (weighted) average correlation of items within the scale [21]. In general, a Cronbach α of 0.7 to 0.8 is regarded as satisfactory for scales to be used as research tools [22]. The distributional characteristics of the DHLI and its subscales were examined to assess normality and detect floor and ceiling effects. Skewness and kurtosis values were used to evaluate score distributions, with values between +1 and –1 interpreted as indicating no or slight nonnormality [23]. Floor or ceiling effects were considered present when more than 15% of participants achieved either the lowest or highest possible score on a subscale [24]. Evidence for construct validity was determined by studying Spearman ρ correlations between total scores on the DHLI 2.0 and sociodemographics, (health-related) internet use, self-reported general health, health care use, and the SILS [19]. We expected the correlations with the other variables to be in line with the correlations of the original DHLI [7]. Additionally, we explored the reliability and validity of an SF DHLI 2.0. By using the “α if item is deleted” option, for each subscale 1 or 2 items were deleted. These short-form subscales were also taken along in the validity analyses.

## Results

### Participants

Table 2 shows the characteristics of the sample. Men (1582/2728, 58%) and respondents with a theory-based educational background (1445/2728, 53%) were overrepresented, that is, people with tertiary education. The mean age of the sample was 60.1 (SD 16.8) years, with a range between 19 and 95 years.

Table 3 presents the sample’s health-related characteristics. A considerable number of participants (1034/2728, 37.9%) reported having at least 1 chronic condition, and about half (1286/2728, 47.1%) had had contact with a medical specialist in the past 12 months. Still, participants scored their own health as rather good on average (7.5 on a scale from 1 to 10).

**Table 2.** Sociodemographics (N=2728).

Characteristic	Value
Gender, n (%)	
Male	1582 (58)
Female	1135 (41.6)
Other	11 (0.4)
Age (y), mean (SD)	60.1 (16.8)
Educational attainment^a^, n (%)	
Low	496 (18.2)
Middle (practice-based/vocational)	718 (26.3)
High (theory-based)	1445 (53)
Missing	69 (2.5)
Family income yearly (gross)^b^	
Less than €15,000	63 (2.3)
€15,000– €31,000	308 (11.3)
€31,000–€38,500	282 (10.3)
€38,500–€46,000	363 (13.3)
€46,000–€77,000	643 (23.6)
€77,000–€92,000	235 (8.6)
€92,000 or more	256 (9.4)
Not applicable/don’t know	514 (18.8)
Missing	64 (2.3)
Personal income monthly (net)	
€1000 or less – €2000	685 (25.2)
€2000–€3000	826 (30.3)
€3000–€4000	547 (20.1)
€4000–€5000	161 (5.9)
€5000 or higher	136 (4)
Not applicable/don’t know	353 (12.9)
Missing	20 (0.7)

a Low: less than primary, primary, and lower secondary education (European qualifications framework 1 (EQF1) and part of EQF2); medium: upper secondary and postsecondary nontertiary education (part of EQF2, EQF3, EQF4, EQF4+); high: tertiary education (EQF5, EQF6, EQF7, EQF8) [25].

b €1=US $1.15.

**Table 3.** Health and health care use.

Variable	Value
Self-reported general health^a^ (N=2625), mean (SD)	7.5 (1.3)
Health care use (N=2728), n yes (%)	
General practitioner	1876 (68.8)
Medical specialist	1286 (47.1)
Mental health care	198 (7.3)
Chronic condition (N=2728), n yes (%)	1034 (37.9)

a On a scale from 1 to 10.

Table 1 provides an overview of participants’ general and health-related internet use. Nearly half of the respondents reported spending approximately 1 to 3 hours online per day. Most participants (1810/2728, 66.4%) rated their own internet skills as (very) good. A large majority had used the internet to search for health-related information (2352/2728, 86.2%), and the use of online portals and digital prescriptions was also relatively high. In contrast, only a small proportion of participants reported using the internet to have a video consultation with their health care provider (428/2728, 15.7%) or to communicate online with fellow patients through a forum (170/2728, 6.2%).

### Reliability and Distributional Properties of the Revised DHLI

Table 4 shows the scores and internal consistency of the DHLI 2.0 items and subscales. Both the FF (24 items) and the SF (16 items) showed strong reliability, with Cronbach α coefficients of 0.94 and 0.90, respectively. Only the protecting privacy full subscale and the using security measures subscale had moderate Cronbach α scores (0.65 and 0.66, respectively).

**Table 4.** Descriptive statistics of digital health literacy instrument (DHLI) 2.0 scales and subscales.

Scale score	N^a^	Value	Cronbach α
Total scale score, mean (SD)			
FF DHLI 2.0^b^ (24 items)	2543	3.17 (0.44)	0.94^c^
SF DHLI 2.0^d^ (16 items)	2728	3.11 (0.48)	0.90^e^
Operational skills, mean^f^ (SD)			
FF 4 items	2728	3.56 (0.51)	0.86
SF 2 items	2728	3.46 (0.64)	0.83
Navigation skills, mean (SD)			
FF 3 items	2436	3.24 (0.54)	0.77
SF 2 items	2436	3.34 (0.58)	0.71
Information searching, mean (SD)			
FF 3 items	2641	3.15 (0.57)	0.89
SF 2 items	2641	3.08 (0.61)	0.86
Evaluating reliability, mean (SD)			
FF 3 items	2520	2.80 (0.66)	0.84
SF 2 items	2520	2.66 (0.73)	0.83
Determining relevance, mean (SD)			
FF 3 items	2453	2.93 (0.61)	0.89
SF 2 items	2453	2.95 (0.63)	0.86
Generating content, mean (SD)			
FF 3 items	1908	3.07 (0.64)	0.93
SF 2 items	1908	3.11 (0.65)	0.91
Protecting privacy, mean (SD)			
FF 3 items	1387	3.52 (0.50)	0.65
SF 2 items	1387	3.68 (0.51)	0.75
Using security measures, mean (SD)			
FF 2 items	2626	3.06 (0.69)	0.66

a Number of respondents per (sub)scale, after excluding missing and “not applicable/do not know” responses.

b FF: full form.

c n=1055; number of respondents that completed at least 16 items, required to compute a total score for the FF.

d SF: short form.

e n=1030; number of respondents that completed at least 8 items, required to compute a total scale score for the SF.

f Possible range between 1 and 4.

Respondents had total mean scores of 3.17 (SD 0.44) and 3.11 (SD 0.48) for the FF and the SF, respectively. Both the FF (skewness = –0.5; kurtosis =0.4) and the SF (skewness = –0.5; kurtosis =0.50) show low skewness and kurtosis values, indicating that their distributions are approximately normal.

The highest scores on the subscales were reported for operational skills (mean 3.6, SD 0.5 and SF mean 3.5, SD 0.6), navigation skills (mean 3.2, SD 0.5 and SF mean 3.3, SD 0.6), and protecting privacy (mean 3.52, SD 0.50 and SF mean 3.68, SD 0.51). This last subscale is skewed for both the full subscale (−1.5) and the short subscale (−2.1), with a ceiling effect where 32.1% (445/1387) scored 4 in the full subscale and 61.9% (858/1387) in the short version, and showed kurtosis in the full subscale (3.8) and in the SF (5.7). Only in the short subscale, operational skills and navigation skills exhibited a skewness of –1, with a ceiling effect, where for operational skills, 49.3% (1345/2728) scored 4 and for navigation skills 26.9% (655/2436). Participants indicated having more difficulties with evaluating reliability (mean 2.8, SD 0.7 and SF mean 2.7, SD 0.7) and with determining relevance of information (mean 2.9, SD 0.6 and SF mean 3.0, SD 0.6). The scores on the new subscale, using security measures, were relatively high for the question regarding 2-factor authentication (mean 3.28, SD 0.78) and low for the question regarding creating and remembering strong passwords (mean 2.83, SD 0.82).

### Construct Validity of the Revised DHLI

Table 5 presents the Spearman ρ correlations between the DHLI 2.0 scale scores, the total scores of both the full and short versions of the DHLI 2.0, and other assessed variables. Looking at sociodemographics, age was moderately negatively correlated with scores on the DHLI 2.0, indicating that older individuals tend to have lower digital health literacy levels. This association was strongest for operational skills and navigation skills. Educational attainment showed a weak positive correlation with the DHLI 2.0, with the highest scores found for generating content, suggesting that educational attainment is somewhat related to digital health literacy skills. Regarding income, family gross income showed a weak positive correlation with the DHLI 2.0, suggesting that a higher income is slightly related to higher digital health literacy skills. Concerning internet use, daily time spent online showed a moderate positive correlation with the DHLI 2.0, suggesting that individuals who spend more time online tend to have higher digital health literacy. Again, the strongest correlations were observed for operational skills. Health-related internet use only weakly correlated with the DHLI 2.0. The DHLI 2.0 showed a moderate positive correlation with SILS, indicating conceptual overlap between the constructs measured by these instruments.

**Table 5.** Spearman ρ correlations between the revised digital health literacy instrument, sociodemographics, (health-related) internet use, health-related variables, and the Single Item Literacy Screener (SILS).

(Sub)scale	Age	Education	Family income	Personal income	Hours spent online	Health-related internet use	General health	Use general practitioner	Use medical specialist	Use mental health care	SILS
Full form (FF) and short form (SF)											
FF 24 items ρ	−0.39	0.22	0.27	0.09	0.32	0.07	0.15	0.09	0.06	−0.02	−0.42
*P* value	<.001	<.001	<.001	<.001	<.001	<.001	<.001	<.001	.002	.27	<.001
SF 16 items ρ	−0.39	0.22	0.26	0.10	0.32	0.12	0.14	0.08	0.05	−0.02	−0.40
*P* value	<.001	<.001	<.001	<.001	<.001	<.001	<.001	<.001	.006	.23	<.001
Operational skills											
FF 4 items ρ	−0.46	0.26	0.28	0.09	0.36	0.14	0.12	0.06	0.07	−0.04	−0.35
*P* value	<.001	<.001	<.001	<.001	<.001	<.001	<.001	.004	<.001	.02	<.001
SF 2 items ρ	−0.47	0.24	0.28	0.09	0.37	0.16	0.11	0.06	0.06	−0.05	−0.34
*P* value	<.001	<.001	<.001	<.001	<.001	<.001	<.001	.001	.004	.006	<.001
Navigation skills											
FF 3 items ρ	−0.35	0.15	0.19	0.05	0.26	0.007	0.1	0.1	0.07	−0.04	−0.34
*P* value	<.001	<.001	<.001	<.001	<.001	.72	<.001	<.001	<.001	.03	<.001
SF 2 items ρ	−0.35	0.15	0.20	0.06	0.26	0.02	0.10	0.09	0.07	−0.03	−0.35
*P* value	<.001	<.001	<.001	.008	<.001	.40	<.001	<.001	<.001	.09	<.001
Information searching											
FF 3 items ρ	−0.26	0.14	0.21	0.09	0.23	0.08	0.13	0.08	0.04	−0.01	−0.31
*P* value	<.001	<.001	<.001	<.001	<.001	<.001	<.001	<.001	.03	.78	<.001
SF 2 items ρ	−0.24	0.12	0.19	0.10	0.22	0.07	0.13	0.07	0.04	−0.00	−0.28
*P* value	<.001	<.001	<.001	<.001	<.001	<.001	<.001	.001	.03	.87	<.001
Evaluating reliability											
FF 3 items ρ	−0.31	0.15	0.17	0.06	0.25	0.06	0.10	0.06	0.05	−0.03	−0.33
*P* value	<.001	<.001	<.001	.005	<.001	.003	<.001	.001	.008	.19	<.001
SF 2 items ρ	−0.26	0.13	0.16	0.07	0.21	0.05	0.10	0.05	0.05	−0.02	−0.30
*P* value	<.001	<.001	<.001	.002	<.001	.02	<.001	.006	.02	.42	<.001
Determining relevance											
FF 3 items ρ	−0.26	0.15	0.17	0.06	0.22	0.08	0.12	0.06	0.01	−0.01	−0.28
*P* value	<.001	<.001	<.001	.005	<.001	<.001	<.001	.002	.56	.72	<.001
SF 2 items ρ	−0.27	0.14	0.17	0.06	0.22	0.07	0.11	0.06	0.01	−0.00	−0.27
*P* value	<.001	<.001	<.001	.006	<.001	.001	<.001	.006	.53	.86	<.001
Generating content											
FF 3 items ρ	−0.13	0.28	0.20	0.09	0.12	0.05	0.13	0.01	−0.01	0.01	−0.34
*P* value	<.001	<.001	<.001	<.001	<.001	.02	<.001	.81	.64	.59	<.001
SF 2 items ρ	−0.15	0.28	0.20	0.09	0.13	0.07	0.12	−0.01	−0.02	−0.01	−0.34
*P* value	<.001	<.001	<.001	.001	<.001	.008	<.001	.81	.30	.74	<.001
Protecting privacy											
FF 3 items ρ	−0.22	0.05	0.05	−0.006	0.13	−0.11	0.05	0.08	0.09	−0.02	−0.28
*P* value	<.001	.09	.09	.83	<.001	<.001	.07	.005	<.001	.41	<.001
SF 2 items ρ	−0.12	0.04	0.06	−0.003	0.06	−0.09	0.03	0.03	0.09	−0.02	−0.20
*P* value	<.001	.11	.03	.91	.03	.001	.30	.20	.002	.52	<.001
Using security measures											
FF and SF 2 items ρ	−0.26	0.19	0.21	0.12	0.24	0.12	0.11	0.06	0.04	−0.01	−0.33
*P* value	<.001	<.001	<.001	<.001	<.001	<.001	<.001	.003	.07	.55	<.001

## Discussion

This paper describes the validation of a revised version of the DHLI, the DHLI 2.0. The original instrument was published in 2017, and 8 years later, it has been updated to better reflect the rapidly evolving digital landscape. Several items were reworded to capture the widespread use of smartphones and tablets. Also, a new 2-item subscale was added to address digital safety, specifically the challenges of choosing and using secure passwords and applying two-step authentication when accessing personalized systems. In our view, the DHLI 2.0 offers a more accurate representation of the competencies required for digital health literacy today.

DHLI 2.0 demonstrated strong reliability, with Cronbach α values that were compared to, and in some cases slightly higher than, those of the original instrument. Construct validity was also adequate, with correlations to sociodemographics, internet use, and general health literacy as expected and consistent with those observed for the original DHLI [7] and in other recent systematic reviews focusing on these relationships [26,27]. A recent review by Wang et al [28] identified DHLIs developed over the past 2 decades, each addressing different aspects of digital health literacy, including the DHLI. Compared to the other included instruments, the DHLI and DHLI 2.0 offer a concise yet theoretically well-grounded instrument [7]. It captures a broad spectrum of skills, ranging from basic operational abilities to higher-order information-processing skills, while also addressing the increasingly important domain of privacy and data security. Moreover, to minimize overestimation bias, common for self-reported skills, items are phrased to assess how easy or difficult respondents find certain tasks or how frequently they encounter difficulties. This validation study also included an SF DHLI 2.0, consisting of 16 items instead of 24. The SF demonstrated good reliability and internal consistency. It facilitates easier administration and allows for integration into larger sets of survey instruments.

In revising the DHLI, we deliberately chose to retain only self-report items and exclude the performance-based tasks that were a unique feature of the original version [7]. Although innovative, those tasks turned out to be too complex to administer and provided only limited added value, mostly in terms of face validity. Furthermore, their applicability was highly dependent on the device, browser, and brand used by respondents (eg, desktop vs mobile), making it nearly impossible to develop a universally valid instrument for use in anonymous research settings. Similar challenges are reported by Crocker et al [29] in their systematic scoping review of performance-based eHealth literacy measures, who also highlight the time-consuming nature of these assessments for both participants and researchers, as well as their equipment requirements. Nevertheless, self-report measures have well-known limitations, and future research could explore the integration of performance-based items into the revised DHLI, also for screening in practice settings.

Some limitations of this study need to be acknowledged. First, validation was conducted through an online questionnaire via a citizen panel. Although our sample was large and broadly representative, the online format likely excluded individuals with the lowest digital skills and contributed to the overrepresentation of higher-educated respondents. The response via the citizen panel from Planbureau Fryslân might have contributed to the overrepresentation of older adults. This may limit the generalizability of the findings, particularly for less educated or younger and middle-aged populations. Validation of the instrument among these currently underrepresented populations is needed in future research to ensure its applicability and reliability across a broad range of people in the population. At present, a study in a patient sample is ongoing, which will provide further insight into the validity of the DHLI 2.0 in a more vulnerable population. Second, the reliability of the scale “Using security measures” did not reach the conventional reliability threshold of α=0.7, which may be due to its coverage of 2 distinct skills (using strong passwords and using 2-step authentication). These items should therefore be considered separately, rather than combined in a sum score, and further research is warranted to determine whether this domain would benefit from the inclusion of additional items. Third, the correlation between DHLI 2.0 scores and health-related internet use was relatively low. This may partly reflect the relatively high mean age of our sample; although many older adults do use the internet regularly for health purposes, they may not possess the full range of skills needed to do so effectively, efficiently, and safely, which is also reported in previous studies [30,31]. Furthermore, motivational factors could influence the relationship, for example, when individuals do not perceive the internet as a good or reliable source of health information and therefore use it less frequently. Finally, health status itself may play a role, as relatively healthy individuals may have limited need for health-related digital tools (eg, patient portals or online communities), despite having adequate or even high levels of digital health literacy [32]. As a final limitation, the total scores of the subscales demonstrated approximately normal distributions, while some subscales exhibited pronounced ceiling effects. The subscale “Protecting privacy” showed the most substantial ceiling effect, potentially influenced by the phrasing of its introductory item: “When you post a message on a public forum or social media…” This condition led to reduced response rates, as only respondents who engage in such online activity reported an answer. Consequently, the responses may reflect a self-selected group with high privacy awareness within the cohort. The ceiling effects in operational and navigation skills might be affected by the online nature of the questionnaire.

The online world and available technologies are changing rapidly. Since the development of the DHLI 2.0, the integration of artificial intelligence into health applications and internet browsers has accelerated significantly. This further stresses the importance of being able to judge the reliability of information, including being aware of its source and the applicability to oneself [33,34]. In this validation study, we specifically see that the skill to evaluate reliability of online information is what many people find challenging. These findings highlight the importance of supporting individuals in developing critical evaluation skills, particularly as digital health environments become more complex and information sources less transparent. This also underscores the need for further research on how to capture the skills required in an increasingly artificial intelligence–driven digital health environment. Future research should also examine the applicability and utility of the DHLI 2.0 among groups at higher risk of limited digital health literacy. Another interesting avenue for future work is to explore the potential of this instrument in practice; this could be within health care, but also within more community- or welfare-oriented organizations. Although not yet validated for that purpose, the DHLI 2.0 (especially the SF) could support decision-making in health consultations, for example, in determining the suitability of specific eHealth tools for patients.

In conclusion, the DHLI 2.0 provides an up-to-date, reliable, valid, and concise measure of digital health literacy, grounded in a strong theoretical framework and covering 8 key domains of digital health literacy. The SF DHLI proves to be a valuable alternative to measure the broad spectrum of digital health literacy skills using 16 items.

## Supplementary material

10.2196/86879 Multimedia Appendix 1 Digital health literacy instrument 2.0 full form.

10.2196/86879 Multimedia Appendix 2 Digital health literacy instrument 2.0 short form.

10.2196/86879 Multimedia Appendix 3 Overview of alterations in DHLI 2.0.

## Abbreviations

DHLI: digital health literacy instrument
DHLI 2.0: revised version of the DHLI
FF: full form
SF: short form
SILS: Single Item Literacy Screener
