# Supplementary material for: Validation of the Updated Digital Health Literacy Instrument and Development of a Short Form: Online Survey Study of the General Population
Source: J Med Internet Res. 2026 Apr 1;28:e86879. doi: 10.2196/86879 (PMC13043908; doi:10.2196/86879)
Supplement: Multimedia Appendix 3 [file jmir-v28-e86879-s003.pdf]

Multimedia Appendix 3

Overview of alterations in DHLI 2.0

Items in original DHLI (Van der Vaart et al, 2017 – see [Journal of Medical Internet Research - Development of the Digital Health Literacy Instrument: Measuring a Broad Spectrum of Health 1.0 and Health 2.0 Skills])	Items in DHLI 2.0 (Van der Vaart et al, 2026).
**1. When using a computer, smartphone, or tablet, how <u>easy or difficult</u> do you find it to...**	
a. use the keyboard (e.g. to type words)?	a. use the keyboard (e.g. to type words)?
b. use the mouse (e.g. to put the cursor in the right field or to click)?	b. use the mouse? (e.g. to click or double click)
c. use the buttons or links and hyperlinks on websites?	c. operate your tablet or smartphone with your fingers (by tapping or swiping)
	d. use the buttons and links or hyperlinks on websites?
**2. When you search the internet for health information, how <u>easy or difficult</u> do you find it to...**	
a. make a choice from all the information you find?	a. choose from the search results? (the list of websites presented by a search engine)
b. use the proper words or search query to find the information you are looking for?	b. come up with the right words/search terms to quickly find the right information in a search engine? (e.g. Google, Yahoo or Bing!)
c. find the exact information you are looking for?	c. find exactly what you are looking for?
d. decide whether the information is reliable or not?	d. decide whether the information is reliable or not?
e. decide whether the information is written with commercial interests? (e.g. by people trying to sell a product?)	e. determine whether the information has (hidden) commercial purposes? (e.g., companies that want to sell a product)
f. check different websites to see whether they provide the same information?	f. check different websites to see if they provide the same information?
g. to decide if the information you found is applicable to you?	g. determine whether the information found applies to you?
h. to apply the information you found in your daily life?	h. apply the information you have found in your daily life?
i. to use the information you found to make decisions about your health (e.g. on	i. to use the information you found to make decisions about your health (e.g. on

nutrition, medication or to decide whether to ask a doctor's opinion)?	nutrition, medication or to decide whether to ask a doctor's opinion)?
**3. When you search for health information on the internet or use a health app, how <u>often</u> does it happen that...**	
a. you lose track of where you are on a website or the internet?	a. you lose track of where you are on a website or in an app?
b. you do not know how to return to a previous page?	b. you do not know how to return to a previous page?
c. you click on something and get to see something different than you expected?	c. you click on something and get to see something different than you expected?
**4. When you write a message about your health, for example on social media, in a health app, or to your family doctor, how <u>easy or difficult</u> do you find it to...**	
a. clearly formulate your question or health-related worry?	a. clearly formulate your questions or concerns about your health in writing?
b. express your opinion, thoughts or feelings in writing?	b. express your opinions, thoughts, or feelings in writing?
c. write your message as such, for people to understand exactly what you mean?	c. write your message in such a way that people understand exactly what you mean?
**5. When you post a message on a public forum or social media, how often...**	
a. do you find it difficult to judge who can read along?	a. do you find it difficult to judge who can read along?
b. do you (intentionally or unintentionally) share your own private information (e.g. name or address)?	b. do you include sensitive personal information in your message? (such as your name or address)
c. do you (intentionally or unintentionally) share someone else's private information?	c. do you include sensitive information about someone else in your message?
**How easy or difficult do you find it to...**	
	a. create and remember strong passwords? (a long password that includes a combination of letters, uppercase letters, numbers, and special characters)
	b. log in to websites (e.g., a patient portal) with two-factor authentication. (that you must verify your identity in two ways, for example with a password and an SMS code)
